# RB Particle Filter Time Synchronization Algorithm Based on the DPM Model

**DOI:** 10.3390/s150922249

**Published:** 2015-09-03

**Authors:** Chunsheng Guo, Jia Shen, Yao Sun, Na Ying

**Affiliations:** 1 College of Communication Engineering, Hangzhou Dianzi University, Hangzhou 310018, China; E-Mails: shenjia_hdu@163.com (J.S.); yingna@hdu.edu.cn (N.Y.); 2 College of Automation Engineering, Hangzhou Dianzi University, Hangzhou 310018, China; E-Mail: sunyao@hdu.edu.cn

**Keywords:** wireless sensor networks, time synchronization, dirichlet process mixture model, rao-blackwellised particle filter

## Abstract

Time synchronization is essential for node localization, target tracking, data fusion, and various other Wireless Sensor Network (WSN) applications. To improve the estimation accuracy of continuous clock offset and skew of mobile nodes in WSNs, we propose a novel time synchronization algorithm, the Rao-Blackwellised (RB) particle filter time synchronization algorithm based on the Dirichlet process mixture (DPM) model. In a state-space equation with a linear substructure, state variables are divided into linear and non-linear variables by the RB particle filter algorithm. These two variables can be estimated using Kalman filter and particle filter, respectively, which improves the computational efficiency more so than if only the particle filter was used. In addition, the DPM model is used to describe the distribution of non-deterministic delays and to automatically adjust the number of Gaussian mixture model components based on the observational data. This improves the estimation accuracy of clock offset and skew, which allows achieving the time synchronization. The time synchronization performance of this algorithm is also validated by computer simulations and experimental measurements. The results show that the proposed algorithm has a higher time synchronization precision than traditional time synchronization algorithms.

## 1. Introduction

Wireless Sensor Networks (WSNs) have broad applications in environmental monitoring, military reconnaissance, health care, and other fields [1,2,3]. Time synchronization is an important step in WSNs, which makes all nodes to coordinate operations in complex sensing tasks [4]. In order to deal with the effects of crystal oscillator frequency deviation and the relative motion between nodes, a random delay modeling and synchronous tracking technology are introduced to improve the accuracy of time synchronization in WSNs, which will benefit the subsequent applications, such as the node location and tracking applications [5,6,7].

Traditional synchronization technologies, such as Network Time Protocol (NTP) or Global Positioning System (GPS) [8,9], is not suitable for WSNs due to their size, cost, and impact on energy consumption. Therefore, many synchronization protocols for WSNs, such as Reference Broadcast Synchronization protocol (RBS) [10], Flooding Time Synchronization Protocol (FTSP) [11], Timing-Sync Protocol for Sensor Networks (TPSN) [12], and Delay Measurement Time Synchronization protocol (DMTS) [13], have been proposed in recent years. Among these protocols, TPSN shows a higher synchronization accuracy and better scalability as the delay is estimated using a two-way information exchange technique. However, non-deterministic delays cause difficulties in continuous clock offset and skew estimation during two-way timing message exchanges, which increase the computational burden of the time synchronization algorithm.

Currently, there are two main methods to solve non-deterministic delays in TPSN—selecting the appropriate delay distribution and calculating the adaptive delay distribution. In the first method, the symmetric exponential Maximum Likelihood (SEML) algorithm or the symmetric Gaussian Maximum Likelihood (SGML) algorithm [14,15,16] estimates clock offset by using Maximum Likelihood Estimation (MLE) in the hypothesis of symmetric exponential or Gaussian delay distributions, respectively. Chaudhari, Serpedin, *et al*. [17] introduced two unbiased estimation methods of clock offset: Best Linear Unbiased Estimation using Order Statistics (BLUS-OS) and Minimum Variance Unbiased Estimation (MVUE). Subsequently, they proved that the unbiased estimation of clock offset also coincides with MLE in the hypothesis of symmetric exponential delays. Obviously, when the application conditions are constantly changing, the clock offset and skew estimation in the determining delay distribution will lack the adaptability. In the second method, the Gaussian Mixture Model (GMM) is often used to estimate the unknown delay distribution; therefore, the estimation results of clock offset can adaptively adjust according to the change in the application conditions. Kim Lee, *et al*. [18,19] proposed estimation algorithms of clock offset based on Gaussian Mixture Kalman Particle Filter (GMKPF), and [20] introduced Iterative Gaussian Mixture Kalman Particle Filter (IGMKPF) in the estimation of clock offset, which improves the robustness of estimation results in arbitrary delay distributions. However, the predetermined number of Gaussian models weakens the flexibility of the delay distribution estimation, and affects the accuracy of time synchronization.

To solve the lack of the adaptive regulation method in modeling the delay distribution based on GMM, we conducted work on two fronts. First, we improve estimation accuracy of delay distributions based on the non-parametric Bayesian model. Next, we reduce the amount of computation in tracking non-Gaussian clock offset and skew based on the Rao-Blackwellised particle filter (RBPF). In order to overcome the limitations of the Gaussian mixture model, the Dirichlet process mixture (DPM) model is used to estimate the delay distribution, which can adjust the number of Gaussian models automatically based on the observational data. Subsequently, the DPM-based Rao-Blackwellised particle filter (DPM-RBPF) algorithm is proposed [21,22], where the system model is divided into linear and non-linear parts—it uses the particle filter and the Kalman filter to estimate the non-linear and the linear parts, respectively. Hence, the DPM-RBPF algorithm can efficiently track clock offset and skew in the non-Gaussian dynamic model. Time synchronization is achieved by improving the efficiency and accuracy of the continuous clock offset and skew estimation process.

The rest of this paper is organized as follows. In Section 2, the DPM-RBPF algorithm is stated in detail—it includes the two-way timing message exchange model, the DPM model description of the observation noise, and the RBPF algorithm. In Section 3, we compare the proposed algorithm with the other algorithms, and verify time synchronization performance of our algorithm. In Section 4, we draw conclusions and look ahead to the development direction of this research.

## 2. RBPF Time Synchronization Algorithm Based on the DPM Model 

The time synchronization is the continuous clock offset and skew estimation, but it is crucial to deal with non-deterministic delay and packet loss. Therefore, this paper proposes a DPM-RBPF algorithm, which introduces a DPM model description of the observation noise and RBPF algorithm based on two-way timing message exchange. Consequently, the clock offset and skew tracking is achieved with the improved computational efficiency and accuracy.

### 2.1. State-Space Equation of Two-Way Timing Message Exchange

**Figure 1:**
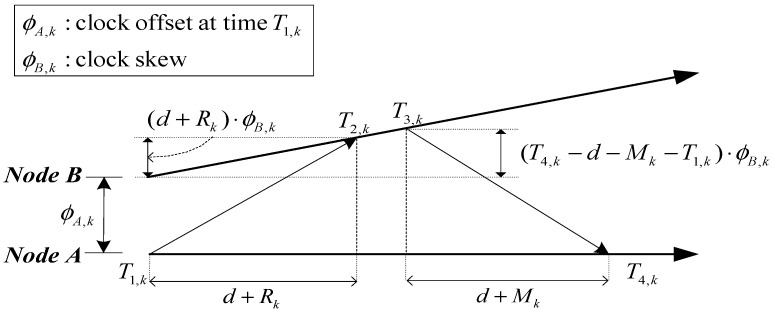
Two-way timing message exchange.

The two-way timing message exchange method is commonly employed in time synchronization. The specific exchange process of this method is illustrated in Figure 1.

In Figure 1, the *k*th time set of message departure and arrival $\text{\{}T_{1,k},T_{2,k},T_{3,k},T_{4,k}\text{\}}$ can be obtained by two-way timing message exchange. $\phi_{A,k}$ represents clock offset of Node A’s clock relative to Node B’s clock at time $T_{1,k}$ in the *k*th exchange process. $\phi_{B,k}$ represents clock skew, $\phi_{B,k}=0$ implies that Node A and Node B have the same clock frequency. d is the fixed transmission delay between Node A and Node B. $R_{k}$ and $M_{k}$ are the non-deterministic network delays from Node A to Node B and from Node B to Node A, respectively. 

Given the above exchange model, the time difference between the uplink and the downlink transmission can be represented as follows:

(1) $$\begin{array}{c} U_{k}=T_{2,k}-T_{1,k}=(d+R_{k})\cdot \phi_{B,k}+\phi_{A,k}\\ V_{k}=T_{4,k}-T_{3,k}=(d+M_{k})\cdot \phi_{B,k}-(\phi_{B,k}-1)(T_{4,k}-T_{1,k})-\phi_{A,k} \end{array}$$

The observation model can be derived from Equation (1) as follows:

(2) $$z_{k}=U_{k}-V_{k}=2\phi_{A,k}+(T_{4,k}-T_{1,k})\cdot \phi_{B,k}+(R_{k}-M_{k})\cdot \phi_{B,k}-(T_{4,k}-T_{1,k})$$

If the clock skew cannot be ignored (*i.e*., $\phi_{B,k}\neq 0$), then the clock offset continue to change with time, as shown in Figure 1. Therefore, the clock offset and skew should be continuously estimated to improve the accuracy and reliability of synchronization. Here, we adopt the constant clock skew model as follows.

(3) $$\phi_{A,k}=\text{Δ}t\cdot \phi_{B,k-1}+\phi_{A,k-1}$$

where Δt is the time interval of two-way timing message exchange, *i.e*., $\text{Δ}t=T_{1,k}-T_{1,k-1}$.

By considering the clock offset $\phi_{A,k}$ and clock skew $\phi_{B,k}$ as a state variable $x_{k}={\left[\phi_{A,k}\;\phi_{B,k}\right]}^{T}$, the state-space equation of two-way timing message exchange model is described as follows:

(4) $$\begin{array}{l} x_{k}=\left[\begin{array}{cc} 1 & \text{Δ}t\\ 0 & 1 \end{array}\right]\cdot x_{k-1}+v_{k-1}\\ z_{k}=\left[\begin{array}{cc} 2 & T_{4,k}-T_{1,k} \end{array}\right]\cdot x_{k}+n_{k} \end{array}$$

where $v_{k-1}$ is the Gaussian state noise with zero mean and covariance Q, which reflects the effects of environmental temperature, crystal ageing, power supply voltage fluctuation, *etc*. $n_{k}=(R_{k}-M_{k})\cdot \phi_{B,k}-(T_{4,k}-T_{1,k})$ is the unknown distribution observation noise. 

If the clock skew can be ignored (*i.e*., $\phi_{B,k}=0$), the initial clock offset is the only parameter to be estimated. Assuming the clock offset as the state variables, the state-space equation of the two-way timing message exchange can be described as follows [18]:

(5) $$\begin{array}{l} x_{k}=x_{k-1}+v_{k-1}\\ z_{k}=2x_{k}+n_{k} \end{array}$$

where $n_{k}=T_{1,k}-T_{4,k}$ is the observation noise. 

### 2.2. Observation Noise DPM Model

**Figure 2:**
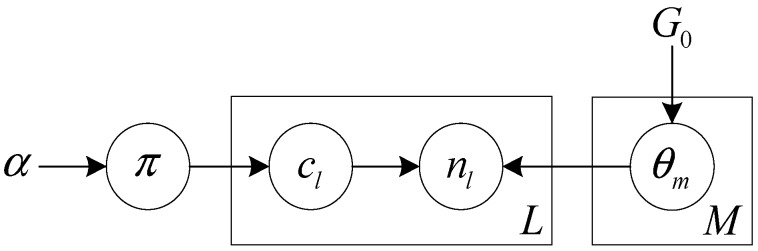
Dirichlet process mixture model.

For the unknown observation noise, the DPM model [23] is used to estimate its probability density function. The DPM model, as a non-parametric Bayesian model, has been widely used in the probability density estimation, data clustering [24], which can flexibly adjust model parameters and the component number. The graphical model of DPM model is shown in Figure 2.

In Figure 2, the observation noise $n_{l}$ obeys the likelihood function F with the indicator $c_{l}$ and the parameter set $\theta =\{\theta_{1},\cdots ,\theta_{m},\cdots ,\theta_{M}\}$, where $\theta_{m}$ is the parameter of the m*th* component posterior based on the prior $G_{0}$ with the parameter $\theta_{0}$. The parameter indicator $c_{l}$ is obtained from a multinomial distribution π with a symmetric Dirichlet prior. Therefore, the formula of the hierarchical structure is expressed as follows:

(6) $$n_{l}\text{ \textasciitilde{} }F\left(n_{l}|\theta_{c_{l}}\right);\;{\text{θ}}_{c_{l}=m}∼\;G_{m};\;c_{l}\sim \;Mul(\text{π});\text{ π }\sim \;Dir\left(\text{α}/M,\cdots ,\text{α}/M\right)$$

where α is the concentration parameter of the Dirichlet distribution. K is the dimensional number of Dirichlet distribution. M is the number of the posterior component.

In this work, the likelihood function F(⋅) is selected to be the Gaussian distribution, and the conjugate prior $G_{0}$ is described as the product of a Gaussian distribution and an inverse chi-squared distribution: $G_{0}(\text{μ},{\text{σ}}^{2}|{\text{θ}}_{0})=𝒩(\text{μ}|{\text{μ}}_{0},{\text{σ}}^{2}/{\text{λ}}_{0})\cdot X^{-1}({\text{σ}}^{2}|{\text{σ}}_{0}^{2},\nu_{0})$, where ${\text{θ}}_{0}{\text{=\{μ}}_{0},{\text{λ}}_{0},{\text{σ}}_{0}^{2},\nu_{0}\}$. ${\text{μ}}_{0}$ and ${\text{λ}}_{0}$ are the mean and the scale parameter of the Gaussian distribution N, respectively. ${\text{σ}}_{0}^{2}$ and $\nu_{0}$ are the variance and the degree of freedom of the inverse chi-squared distribution $X^{-1}$, respectively. Therefore, the posterior distribution is also a normal inverse chi-squared distribution. If the observation noise set $n=\{n_{1}^{m},n_{2}^{m},\ldots ,n_{L_{m}}^{m}\}$ is received, where all $L_{m}$ observation noise have the indicator $c_{l}=m$, the m*th* component posterior is expressed as following:

(7) $$\begin{array}{l} p(\text{μ},{\text{σ}}^{2}{\text{|θ}}_{m})=N(\text{μ}|{\text{μ}}_{m},{\text{σ}}^{2}/{\text{λ}}_{m})\cdot X^{-1}({\text{σ}}^{2}|{\text{σ}}_{m}^{2},\nu_{m})\\ \text{ where }{\text{μ}}_{m}=\left({\text{λ}}_{0}{\text{μ}}_{0}+\sum_{i=1}^{L_{m}}n_{i}^{m}\right)/\left({\text{λ}}_{0}+L_{m}\right),\\ \;{\text{λ}}_{m}={\text{λ}}_{0}+L_{m},\;\nu_{m}=\nu_{0}+L_{m},\\ \;{\text{σ}}_{m}^{2}={\text{σ}}_{0}^{2}+\sum_{i=1}^{L_{m}}{\left(n_{i}^{m}\right)}^{2}+{\left({\text{λ}}_{0}{\text{μ}}_{0}/\sqrt{{\text{λ}}_{0}}\right)}^{2}-{\left(\left({\text{λ}}_{0}{\text{μ}}_{0}+\sum_{i=1}^{L_{m}}n_{i}^{m}\right)/\sqrt{{\text{λ}}_{0}+L_{m}}\right)}^{2} \end{array}$$

If the observation noise $n_{l}$ is randomly selected, the indicator $c_{l}$ and the parameter of the component posterior ${\text{θ}}_{m}{\text{=\{μ}}_{m},{\text{λ}}_{m},{\text{σ}}_{m}^{2}\text{,}\nu_{m}\text{\}}$ can be updated. Following the Chinese restaurant scheme of the Dirichlet process [21], the m*th* component posterior is formulated as follows:

(8) $$\begin{array}{l} \;p(c_{l}=m|c_{-l},n_{k},\text{α},{\text{θ}}_{m})\propto p(c_{l}=m|c_{-l},\text{α})p(n_{k}|c_{l}=m,{\text{θ}}_{m})\\ p(c_{l}=m|c_{-l},\text{α})=\{\begin{array}{c} L_{-l,m}/(L+\text{α}-1\text{) if }m\text{ has been appeared }\\ \text{α}/(L+\text{α}-1\text{) if }m\text{ is new } \end{array} \end{array}$$

where the indicator set $c_{-l}$ is defined as $\left\{c_{1},\ldots ,c_{l-1},c_{l+1},\ldots ,c_{L}\right\}$. L denotes the number of indicators. $L_{-l,m}$ is the number of the indicator, which is equal to m in the indicator set $c_{-l}$.

The likelihood function $p(n_{k}|c_{l}=m,{\text{θ}}_{m})$ is calculated as follows:

(9) $$\begin{array}{l} p(n_{k}|c_{l}=m,\theta_{m})=\int_{\text{μ}}\int_{{\text{σ}}^{2}}p(n_{k}|\text{μ},{\text{σ}}^{2})p(\text{μ},{\text{σ}}^{2}|\theta_{m})d\text{μ}d{\text{σ}}^{2}\\ {\text{ =π}}^{-1/2}{\left(\frac{{\text{λ}}_{m}+1}{{\text{λ}}_{m}}\right)}^{-1/2}{\left(\frac{{\text{σ}}_{m}^{2}+n_{k}^{2}+\frac{\left({\text{λ}}_{m}+1\right)S_{m}^{2}-\lambda_{m}{\left(S_{m}+n_{k}\right)}^{2}}{\lambda_{m}\left(\lambda_{m}+1\right)}}{{\text{σ}}_{m}^{2}}\right)}^{-(\nu_{m}+1)/2}\frac{\Gamma \left(\frac{\nu_{m}+1}{2}\right)}{\Gamma \left(\frac{\nu_{m}}{2}\right)} \end{array}$$

where $S_{m}={\text{λ}}_{0}{\text{μ}}_{0}+sum_{m}$, $sum_{m}$ represents the sum of observation noise, whose indicator is equal to m. 

After the iterative update of the indicator and the parameter of the component posterior, the concentration parameter α is also sampled from the posterior distribution. Assume α~Gamma(a,b), a gamma prior with shape a>0 and scale b>0. In this paper, we adopt the auxiliary variable method for sampling α. By defining auxiliary variables w and s, where w is a variable with continuous values in [0,1], and s is a binary {0,1} variable, the component posterior p(α|w,s,m) is expressed as [25]:

(10) $$p\left(\text{α}|w,s,m\right)\propto {\text{α}}^{a-1+m-s}e^{-\text{α}\left(b-\log w\right)}$$

which is a gamma distribution with parameters a+m−s and b−logw. m (m>1) is the number of normal components in the mixture model, after given α, the w, s and m may be sampled from the following distribution is given by:

(11) $$\begin{array}{l} p(w|\text{α})\propto w^{\text{α}}{\left(1-w\right)}^{L-1}\\ p(s|\text{α})\propto (L/\text{α}{)}^{s}\\ p(m|\text{α},L)\propto s(L,m){\text{α}}^{m}\Gamma (\text{α})/\Gamma (\text{α}+L) \end{array}$$

where w and s obey beta and binomial distributions, respectively. The s(L,m) and Γ(⋅) are unsigned Stirling numbers of the first kind and gamma function, respectively. The number of normal components m can be calculate based on the concentration parameter α and the number of observations L, it means that the DPM model can flexibly adjust the number of gaussian components using the observation data.

### 2.3. State Tracking Based on DPM-RBFPF

For the non-Gaussian observation noise in the state-space equation of clock offset or skew, the DPM-RBPF algorithm is used for time synchronization. The DPM model is used to describe the observation noise. RBPF is used to track clock offset or skew. RBPF is widely used in visual tracking, simultaneous localization and mapping (SLAM), and other fields [26,27]. It divides the state variables into linear and non-linear parts based on the Rao-Blackwell statistical theory, and improves the computational efficiency of the particle filter by integrating the Kalman filter.

As shown in Equation (4), by exchanging the times information between Node A and B for L times, we get the entire observation data $z_{k}=T_{2,k}+T_{3,k}-T_{1,k}-T_{4,k}$. The clock offset and skew defined as the state variables $x_{k}$, and the indicator of the model component $c_{k}$ is defined as the hidden variable. According to Bayes’ theorem, the posterior probability density function $p(x_{k},c_{k}|\alpha ,\theta ,z_{1:k},x_{1:k-1})$ for the unknown variables $\left[x_{k},c_{k}\right]$ can be decomposed into the linear section $p(x_{k}|c_{k},\theta ,z_{k},x_{k-1})$ and the non-linear section $p(c_{k}|\alpha ,\theta ,z_{k},x_{k-1})$ shown as follows:

(12) $$p(x_{k},c_{k}|\text{α},\theta ,z_{1:k},x_{1:k-1})=p(x_{k}|c_{k},\theta ,z_{k},x_{k-1})\cdot p(c_{k}|\text{α},\theta ,z_{k},x_{k-1})$$

Thus, the estimation of the unknown variables is divided into the estimation of the linear and non-linear variables independently through the marginalization of the probability density function. The Kalman filter is used to estimate the linear variable, and the particle filter is used to calculate the non-linear variable. Since the variable dimension of $p(c_{k}|\text{α},\theta ,z_{k},x_{k-1})$ is less than the variable dimension of $p(x_{k},c_{k}|\text{α},\theta ,z_{k},x_{k-1})$, the DPM-RBPF algorithm needs fewer particles than the standard particle filter, which can effectively improve the computational efficiency of this algorithm [28]. The following section describes linear and non-linear estimation, respectively.

The posterior probability density of the non-linear hidden variable $c_{k}$ can be approximated by a set of random particles with certain importance weight, such that the posterior $p(c_{k}|\text{α},\theta ,z_{k},x_{k-1})$ can be approximated as follows:

(13) $$p(c_{k}|\text{α},\theta ,z_{k},x_{k-1})=\sum_{i=1}^{N}w_{k}^{i}\cdot \text{δ}\left(c_{k}-c_{k}^{i}\right)$$

where $c_{k}^{i}$ is the $i^{th}$ particles with the importance weight $w_{k}^{i}$, δ(⋅) is the Dirac delta function. N is the number of particles.

Initially, the particle $c_{k}^{i}$ is sampled according to the Equation (8), and its importance weight $w_{k}^{i}$ is calculated using the sequential Monte Carlo sampling as follows:

(14) $$w_{k}^{i}=w_{k-1}^{i}\frac{p(z_{k}|c_{k},\theta ,z_{k-1},x_{k-1})\cdot p(c_{k}|\theta ,z_{k-1},x_{k-1})}{q(c_{k}|\theta ,z_{k},x_{k-1})}\;i=1,2,\ldots ,N$$

In order to simplify the calculation of the importance weight $w_{k}^{i}$, the proposal distribution $q(c_{k}|\theta ,z_{k},x_{k-1})$ is usually chosen to be equal to the conditional posterior $p(c_{k}|\theta ,z_{k-1},x_{k-1})$. Hence, the importance weight can be updated by using the formula $w_{k}^{i}=w_{k-1}^{i}p(z_{k}|c_{k},\theta ,z_{k-1},x_{k-1})$. In a specific implementation process, the particles need to be re-sampled to prevent the degradation of particles, according to the weighting coefficient.

After the nonlinear hidden variable $c_{k}$ is sampled using the PF method, the linear state variable $x_{k}$ is calculated by the Kalman filter method. For each particle $c_{k}^{i}$, we can recursively calculate the state variable $x_{k}^{i}$ and the variance $P_{k}^{i}$ by using a Kalman filter as shown below:

(15) $$\left(x_{k}^{i},P_{k}^{i}\right)=KF\left(c_{k}^{i},\theta ,x_{k-1}^{i},P_{k-1}^{i},z_{k}\right)$$

Therefore, the posterior probability density function $p(x_{k}|c_{k},\theta ,z_{k},x_{k-1})$ is approximately expressed by using a mixture of densities shown as follows:

(16) $$p(x_{k}|c_{k},\theta ,z_{k},x_{k-1})=\sum_{i=1}^{N}w_{k}^{i}\;\cdot \text{δ}\left(x_{k}-x_{k}^{i}\right)$$

Finally, the minimum mean squared error (MMSE) estimation of linear state variable and noise covariance matrix can be written as follows:

(17) $$\begin{array}{l} {\hat{x}}_{k}=\sum_{i=1}^{N}w_{k}^{i}x_{k}^{i}\\ {\hat{P}}_{k}=\sum_{i=1}^{N}w_{k}^{i}\left[P_{k}^{i}+\left(x_{k}^{i}-{\hat{x}}_{k}\right){\left(x_{k}^{i}-{\hat{x}}_{k}\right)}^{T}\right] \end{array}$$

### 2.4. Algorithmic Process 

We propose the DPM-RBPF algorithm to improve the accuracy and efficiency of continuous clock offset and skew estimation in TPSN. It includes three key steps. First, the state-space equation is initialized and the DPM model is trained based on the approximate observation noise in Equation (5). Next, the particles of the indicator are re-sampled based on the updated weights. Furthermore, the DPM model is continuously retrained based on the new obtained observations and state values. Finally, the particles of the state variable are calculated using the Kalman filter and the state variable is estimated to complete time synchronization between two nodes. The process of DPM-RBPF algorithm is listed as shown below in Algorithm 1:
**Algorithm 1.** DPM-RBPF algorithm.Step (1) Initialize the DPM model and the state-space equation.
•Set the concentration parameter α and the base distribution $G_{0}$. According to the approximate observation noise data set, the DPM model is trained using Equations (7)–(11).•Set the initial state variable ${\hat{x}}_{0}={\hat{x}}_{ML}$.•Sample the N initial particles of the model component indicator from DPM model.•Initialize the importance weight of particles uniformly.Step (2) Resample the particles and update the DPM model.
•Calculate and normalize the importance weights using Equation (14).•Generate a new set of particles ${\left\{c_{k}^{i}\right\}}_{i=1}^{N}$ with the corresponding importance weights ${\left\{w_{k}^{i}\right\}}_{i=1}^{N}$ using the resampling method.•DPM model is updated based on the observations and estimated state variables in a time interval.
Step (3) Estimate the state variable.
•According to Equation (15), the state variables of all particles are updated. According to the Equation (17), the state variables are calculated.•If the online calculation is not complete, return to Step (2).

## 3. The Performance and the Analysis

In this section, the estimation accuracy and computational efficiency of the DPM-RBPF algorithm are compared with the Maximum Likelihood Estimation (MLE) [15,16], the Advanced Self-Correcting Time Synchronization (ASCTS) [29] and the Iterative Gaussian Mixture Kalman Particle Filter (IGMKPF) algorithm [20]. The performance of the proposed algorithm is verified via computer simulations and experimental measurements, and the influence of the number of observations, particles, and other parameters is analyzed in detail. Unless otherwise specified in the following experiments, it is assumed that the variance Q of state noise is equal to $1e^{-4}$, the number of the indicator particles is set to 500, and the concentration parameter α is followed by gamma prior with the shape a=1 and the scale b=1.

### 3.1. Simulations’ Comparison

**Figure 3:**
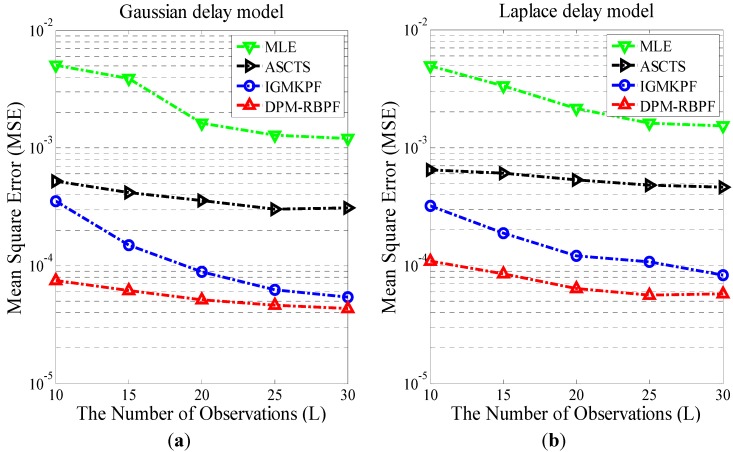
(**a**) MSE of clock offset estimators for symmetrical Gaussian delay; (**b**) MSE of clock offset estimators for symmetrical exponential delay.

Assuming that the non-deterministic delay model obeys Gaussian distribution and Laplace distribution, time synchronization results of MLE, ASCTS, IGMKPF and DPM-RBPF algorithm are compared. For the Gaussian distribution experiment, the parameters are set as follows: the non-deterministic delay $R_{k}$ and $M_{k}$ are assumed to follow the Gaussian distribution with zero mean and 0.01 variance. The initial value of the state ${\hat{x}}_{0}$ is set to 0.001. The average results of 30 trials of the three algorithms are shown in Figure 3a. The vertical coordinate is the mean square error (MSE), which is used to evaluate the performance of the clock offset and skew estimation. The horizontal coordinate is the number of two-way timing message exchanges. Each experiment was performed 30 times two-way timing message exchange to track the clock offset and skew. In the experiment of Laplace distribution, the parameters are set as follows: $R_{k}$ and $M_{k}$ are assumed to follow the Laplace distribution with zero location parameter and 0.01 scale parameter. The other settings are identical to the experiment of Gaussian distribution. The average results are shown in Figure 3b.

As shown in Figure 3, with an increase in the number of observations, there is a gradual reduction in MSE. A smaller MSE implies that there is a higher precision of time synchronization. Comparing the four algorithms—MLE, ASCTS, IGMKPF and DPM-RBPF—DPM-RBPF is better than MLE, ASCTS and IGMKPF in MSE of the clock offset estimation. This indicates that the DPM model describes the observation noise more accurately. The hypothetical observation noise distribution model used by MLE is probably different from the actual observation noise distribution model. Therefore, the performance of MLE is worse than the other two algorithms, which implement an adaptive observation noise model.

**Figure 4:**
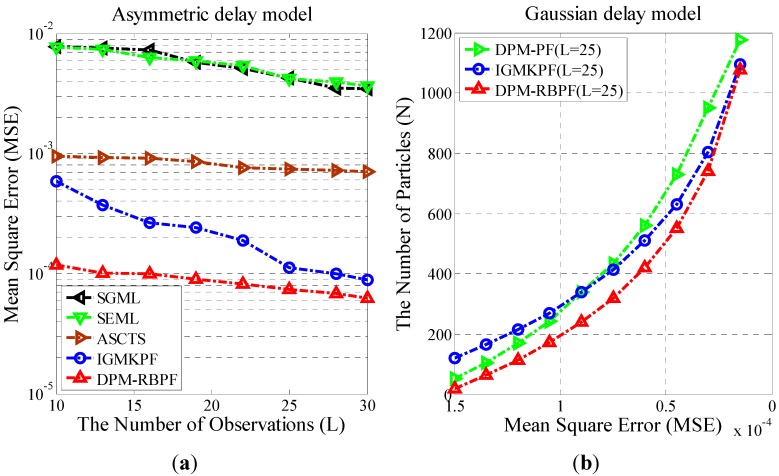
(**a**) MSE of clock offset estimators for mixing of a Gaussian and an exponential; (**b**) The relationship between the number of particles and MSE.

Gaussian distribution and Laplace distribution are symmetrical, but the delay noise is often asymmetric. As a result, the performances of the algorithms are compared under conditions of asymmetric delay noise, and the computational efficiency of DPM-RBPF and IGMKPF are tested. Assuming the non-deterministic delay model obeys an asymmetric distribution, $R_{k}$ is the Gaussian distribution with zero mean and 0.01 variance, and $M_{k}$ is the exponential distribution with 0.01 rate parameter. Figure 4a shows the comparison of the estimation results of different algorithms. Since the hypothetical noise distribution is inconsistent with the actual noise distribution, the performance of MLE under conditions of symmetrical Gaussian distribution or symmetrical exponential distribution (Laplace distribution) becomes worse. The performance of DPM-RBPF remains superior to the four comparative algorithms. Figure 4b shows the number of particles needed by DPM-RBPF, DPM-PF and IGMKPF under different MSE. For the same MSE, DPM-RBPF requires fewer particles than DPM-PF and IGMKPF. The calculation extent of DPM-RBPF is less than the other algorithms to achieve the same accuracy. This means that the RBPF can improve the computational efficiency.

### 3.2. Parameters’ Analysis

The impact of the relevant parameters on the DPM-RBPF algorithm is discussed in this section. Since sensor nodes are energy-constrained, the number of the time message exchanges between the nodes is limited, and the number of particles for the RBPF algorithm is also limited. In order to minimize energy consumption for a given synchronization accuracy, we evaluate the impart of the number of observations and the number of particles on the DPM-RBPF algorithm.

#### 3.2.1. Relationship Analysis between the Number of Observations and MSE

**Figure 5:**
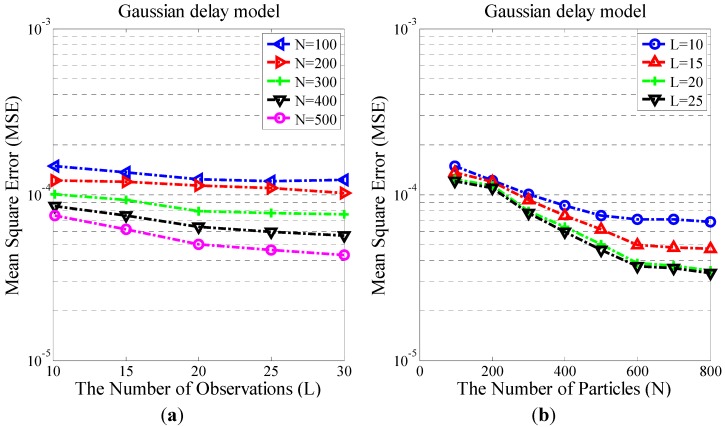
(**a**) The relationship between the Number of Observations and MSE; (**b**) The relationship between the Number of Particles and MSE.

Figure 5a shows the relationship between the number of observations L and the MSE of the clock offset when the numbers of particles are 100, 200, 300, 400, and 500, respectively. As can be seen from the figure, with fewer particles (N = 100 or N = 200), MSE only changes slightly with an increase in the number of observations. In other words, when the number of particles is too small, the DPM-RBPF algorithm cannot fully exploit the time information of observations. However, when the number of particles is greater than 300, we find that the MSE becomes smaller with an increase in the number observations. In order to guarantee that MSE is less than $7e^{\text{-}5}$, and to minimize energy consumption of nodes, we select L=20 and N≥400.

#### 3.2.2. Relationship Analysis between the Number of Particles and MSE

Figure 5b shows the relationship of the number of RB particle filter and the MSE when the number of observations is 10, 15, 20, and 25, respectively. From the figure, we can see that when the number of observations is constant, MSE increases as the number of particles reduces, and vice versa. The two curves L=20 and L=25 substantially coincide and MSE is similar. When the number of particles is larger than 600, MSE stabilizes. Considering synchronization accuracy and energy consumption comprehensively, we finally select L=20 and N=500.

### 3.3. Experimental Measurement

**Figure 6:**
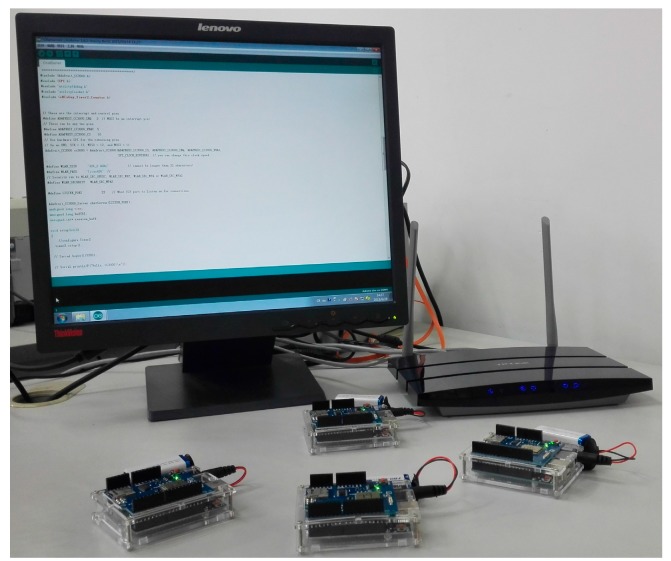
The experimental platform with four nodes (Arduino Uno board).

To evaluate the time synchronization performance, we set up a testing system with four mobile nodes based on Arduino Uno boards, as shown in Figure 6. Arduino Uno is a microcontroller board based on the ATmega328 processor with 16 MHz crystal clock oscillator. The on-chip memory includes 32 KB of flash memory, 1 KB of EEPROM and 2 KB of SRAM. All Arduino Uno configured with CC3000 WiFi Shield establish the communication links to each other through a wireless router. The program of the proposed DPM-RBPF algorithm is running on the control computer with a 2.27 GHz CPU and 16 GB RAM. 

According to the two-way timing message exchange method, the control computer periodically sends the timestamps packet to four mobile nodes. When the mobile node receives a timestamps packet, it would read the clock at the time of receiving and sending the timestamps packet. These clocks are calibrated based on the clock offset in the timestamps packet, and fill back the timestamps packet. Then, the timestamps packet was immediately sent back to the control computer for the next clock skew tracking. The format of timestamps packet is {*T_0,k_,T_1,k_,T_2,k_,T_3,k_,T_4,k_*}, which includes the clock offset *T_0,k_*, the computer clock *T_1,k_* and *T_4,k_* at time of receiving and sending the timestamps packet, and the node clock *T_2,k_* and *T_3,k_* at the time of receiving and sending timestamps packet, where *k* is the number of two-way timing message exchanges. If there is no clock offset calibration (It means the *T_0,k_* in timestamp packet is set to 0), the clock in timestamps packet from *k_0_* to *k_0_* + 3 times is shown in the left section of Table 1. Due to the existence of the clock offset and skew between the control computer and the mobile node, *T_1,k_, T_4,k_* and *T_2,k_ , T_3,k_* have a significant difference. If there is a clock offset calibration, as shown in the right section of Table 1, the clock values from *T_1,k_* to *T_4,k_* continue to increase, which is consistent with the chronological laws of the timestamps packet exchange.

**Table 1:** The comparison between no calibration and calibration (time interval 2 s).

*k*	No calibration					Calibration (µs)				
	*T_0,k_*	*T_1,k_*	*T_2,k_*	*T_3,k_*	*T_4,k_*	*T_0,k_*	*T_1,k_*	*T_2,k_*	*T_3,k_*	*T_4,k_*
*k_0_*	0	118,104,732	100,814,673	100,816,003	118,225,238	277,031	118,104,732	118,161,017	118,162,347	118,225,238
*k_0_ + 1*	0	120,234,711	102,616,610	102,617,649	120,306,343	275,900	120,234,711	120,238,854	120,239,893	120,306,343
*k_0_ + 2*	0	122,324,748	104,408,959	104,410,527	122,395,988	307,936	122,324,748	122,339,139	122,340,707	122,395,988
*k_0_ + 3*	0	124,414,626	106,189,262	106,190,567	124,564,677	314,981	124,414,626	124,434,423	124,435,728	124,564,677

**Figure 7:**
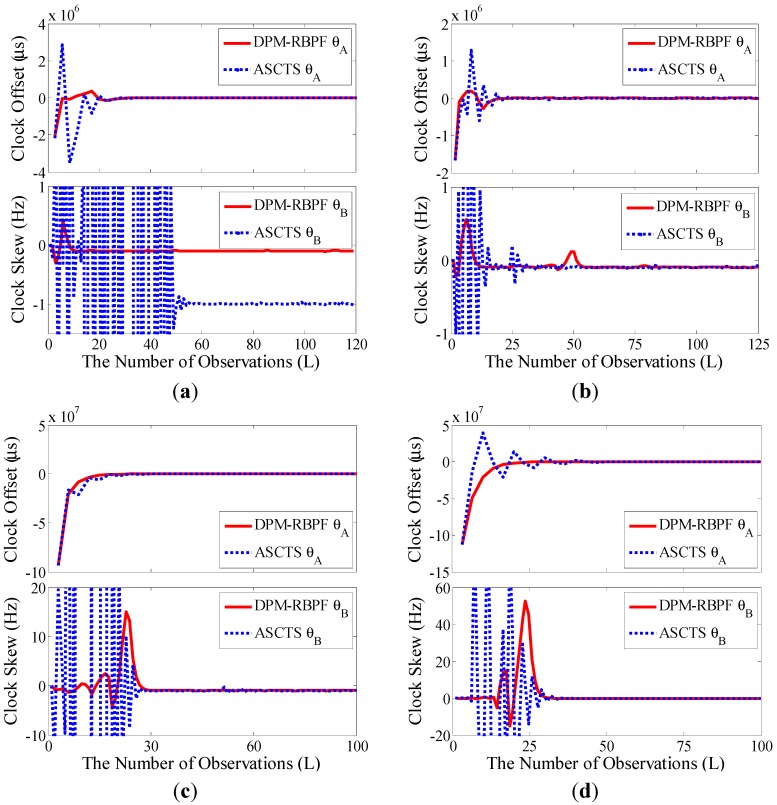
Tracking curves of ASCTS and DPM-RBPF (time interval 5 s). (**a**) Node A; (**b**) Node B; (**c**) Node C; (**d**) Node D.

Using the build test system, the clock synchronization performances of ASCTS and DPM-RBPF algorithm were compared. The tracking curves of the clock offset and skew of four mobile nodes are shown in Figure 7. There is a certain clock offset and skew to every mobile node at the start time. Through tracking the clock offset and skew, the clock offset gradually moves towards zero, which means the clock synchronization can be achieved. Compared with ASCTS, DPM-RBPF can quickly complete the clock synchronization, which calibrates the clock offset at 20, 22, 25, 39 steps, respectively, to four mobile nodes. The tracking curves of DPM-RBPF algorithm show a small fluctuation. This means that the proposed method has better tracking performance.

Quantitative analysis of time synchronization is shown in Table 2. The mean and standard deviation of clock offset and skew in the steady state are listed. Since the observation noise is randomly distributed with nonzero mean, the estimation of clock offset in steady state has certain bias. This bias can usually be counteracted by system calibration. The standard deviation (STD) in Table 2 shows less tracking fluctuation in DPM-RBPF than ASCTS, which means that the time synchronization performance of DPM-RBPF is superior to ASCTS’s.

**Table 2:** Quantitative analysis of time synchronization in steady state (time interval 5 s).

	Node A		Node B		Node C		Node D	
	MEAN	STD	MEAN	STD	MEAN	STD	MEAN	STD
ASCTS θ_A_(µs)	−53.9	408.4	−33.5	313.7	−25.4	552.3	18.3	319.5
ASCTS θ_B_(Hz)	−0.99	0.004	−0.101	0.003	−0.99	0.004	−0.99	0.003
DPM-RBPF θ_A_(µs)	16.1	288.8	160.2	211.5	151.3	379.3	36.4	260.5
DPM-RBPF θ_B_(Hz)	−0.10	0.002	−0.099	0.002	−0.99	0.001	−0.99	0.002

**Figure 8:**
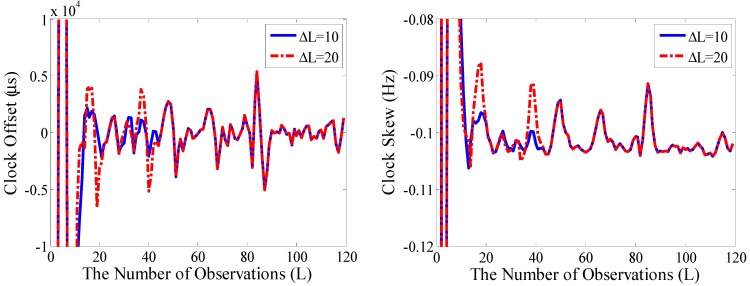
Tracking curves for different intervals (time interval 5 s).

In order to reveal the effect of the DPM model updated in the experiment, different observation intervals ΔL are selected. It means the DPM model is updated after obtaining a different number of observation noises, which are calculated based on the observations and the estimated state variables. When two different time intervals 10 and 20 are selected, the tracking curves of the clock offset and skew on node A are shown in Figure 8.

As shown in Figure 8, since the update of the observation noise model is inconsistent, the tracking curves appear different within the 10–40 observation point range. Despite ignoring the clock skew in the initialization of the observation noise model, the tracking curves soon become identical after more than 40 points. It means that the updating DPM model can better adapt to environmental change and the DPM model is able to describe the unknown observation noise, which ensures the accurate tracking of clock offset and skew.

## 4. Conclusions

In this paper, we propose a novel time synchronization algorithm, DPM-RBPF, to improve the accuracy and efficiency of continuous clock offset and skew estimation in a dynamic sensor network. In the proposed algorithm, a unified state-space equation is constructed to track the clock offset and skew, and a DPM model is used to describe the distribution of observation noise, which can flexibly adjust the number of Gaussian components to enhance the adaptability of the observation noise model. In addition, simulation experiments indicate the advantages of the proposed algorithm in terms of estimation precision and computational efficiency compared with the other algorithms—such as SGML, SEML, ASCTS and IGMKPF algorithms. We also discuss the parameters’ selection process of the proposed algorithm. The results of computer simulations and test systems demonstrate that the proposed algorithm outperforms other algorithms in time synchronization accuracy.
